# Silk Fibroin and Pomegranate By-Products to Develop Sustainable Active Pad for Food Packaging Applications

**DOI:** 10.3390/foods10122921

**Published:** 2021-11-25

**Authors:** Marta Giannelli, Valentina Lacivita, Tamara Posati, Annalisa Aluigi, Amalia Conte, Roberto Zamboni, Matteo Alessandro Del Nobile

**Affiliations:** 1 Consiglio Nazionale delle Ricerche, Istituto per la Sintesi Organica e la Fotoreattività (CNR-ISOF), Via Piero Gobetti 101, 40129 Bologna, Italy; marta.giannelli@isof.cnr.it (M.G.); tamara.posati@isof.cnr.it (T.P.); annalisa.aluigi@isof.cnr.it (A.A.); roberto.zamboni@isof.cnr.it (R.Z.); 2 Department of Agricultural Sciences, Food and Environment, University of Foggia, Via Napoli 25, 71121 Foggia, Italy; valentina.lacivita@unifg.it (V.L.); matteo.delnobile@unifg.it (M.A.D.N.)

**Keywords:** bio-based plastic, silk fibroin, active packaging, pomegranate by-products

## Abstract

In this study, a bio-based polymeric system loaded with fruit by-products was developed. It was based on silk fibroin produced by the silkworm *Bombyx mori* and pomegranate peel powder, selected as active agent. The weight ratio between fibroin and pomegranate powder was 30:70. Pads also contained 20% *w*/*w* of glycerol vs. fibroin to induce water insolubility. Control systems, consisting of only fibroin and glycerol, were produced as reference. Both control and active systems were characterized for structural and morphological characterization (Fourier-transform infrared spectroscopy and optical microscope), antioxidant properties and antimicrobial activity against two foodborne spoilage microorganisms. Results demonstrate that under investigated conditions, an active system was obtained. The pad showed a good water stability, with weight loss of about 28% due to the release of the active agent and not to the fibroin loss. In addition, this edible system has interesting antioxidant and antimicrobial properties. In particular, the pad based on fibroin with pomegranate peel recorded an antioxidant activity of the same order of magnitude of that of vitamin C, which is one of the most well-known antioxidant compounds. As regards the antimicrobial properties, results underlined that pomegranate peel in the pad allowed maintaining microbial concentration around the same initial level (10^4^ CFU/mL) for more than 70 h of monitoring, compared to the control system where viable cell concentration increased very rapidly up to 10^8^ CFU/mL.

## 1. Introduction

Fruit and vegetables produce enormous amounts of waste and therefore greatly contribute to soil and water pollution. It is nevertheless true that a significant proportion of them are by-products (leaves, peel, seeds, pulp, etc.) generated from processing units. The by-products are rich in valuable bioactive compounds, such as either simple sugars, carbohydrates, polysaccharides, pectin and fibers, phytochemical, antioxidants, antimicrobials, phenolic compounds, including vitamin C and E, flavonoids, carotenoids such as lycopene, or in combination [1]. In order to overcome their impact, industrial ecology concepts have been developed with the aim of using these by-products as raw materials for new food applications [2,3]. Several recent researches summarize the potential uses of by-products as low-cost food ingredients to enhance food properties or to prolong their shelf life [4,5,6,7]. Starting from this perspective, another possible strategy for turning the problem of fruit or vegetable waste management into an opportunity, proposed the waste’s potential use for the development of active food packaging solutions [8,9,10]. In this context, considerable research is focusing particular attention to pomegranate by-products in terms of peel, seed and pomace, deriving from pomegranate processing. The reasons can be ascribed to high scavenging properties of free radicals, antimicrobial and anti-mutagenic properties of compounds contained in pomegranate by-products or in the extracts [11,12,13]. Recently, Kandylis and Kokkinomagoulos [14] reviewed food applications and potential health benefits of pomegranate and its derivatives, due to important proximate composition, mineral content, antibacterial and antifungal activity. Despite the numerous food applications of pomegranate by-products and derived extracts to improve nutritional quality or prolong shelf life [15,16,17,18,19], the literature dealing with specific reutilization of pomegranate peel powder in food packaging is still not very abundant, giving rise to belief that more exciting progress will be made in addressing these challenges in the future. Hanani et al. [20,21], in two different studies, proposed the adoption of pomegranate peel powders to develop active packaging systems starting from fish gelatin or gelatin/polyethylene. Even though the studies revealed films with acceptable physical properties and interesting antimicrobial and antioxidant performance, some criticisms appeared above all in terms of increased water vapor permeability. To address these problems, other authors incorporated pomegranate peel extracts in the polymeric materials and applied the films with success to dairy products, orange fruit, meat and fish-based food, thus demonstrating the efficacy on shelf-life prolongation of packaged products [22,23,24,25,26]. However, in the perspective of sustainability, the approach to recycle by-products, further than the sole peel extract, is considered more useful for reducing the costs of waste disposal, following the principle of the circular economy.

It is also widely recognized that environmental impact generated by the petroleum-derived polymers is pushing the development of greener alternative and therefore, ideal candidates for novel active packaging are materials from renewable and biodegradable sources. In this context, one of the most interesting bio-based materials is silk fibroin (SF), produced by the silkworm *Bombyx mori* (*B. mori*), being a natural and edible protein. SF is largely employed to obtain protein-based-biomaterials of potential interest in biomedical and tissue engineering fields [27,28,29]. Regenerated SF water-based solution can be processed in several forms including micro and nanofibers, nanoparticles, films and sponges [30,31,32]. Among these forms, SF film has found application in high technological devices and for the development of eco-sustainable, biocompatible and biodegradable substrates [33,34]. Recently, Marelli et al. [35] developed an SF coating able to modulate gas diffusion of food packaging, with the aim to maintain food freshness. Furthermore, fibrous silk waste (obtained from rejected cocoons) has also been proven to be promising raw materials to obtain aqueous SF solutions, used as edible and transparent coating for fresh food with high detrimental kinetics [36].

**Figure 1:**
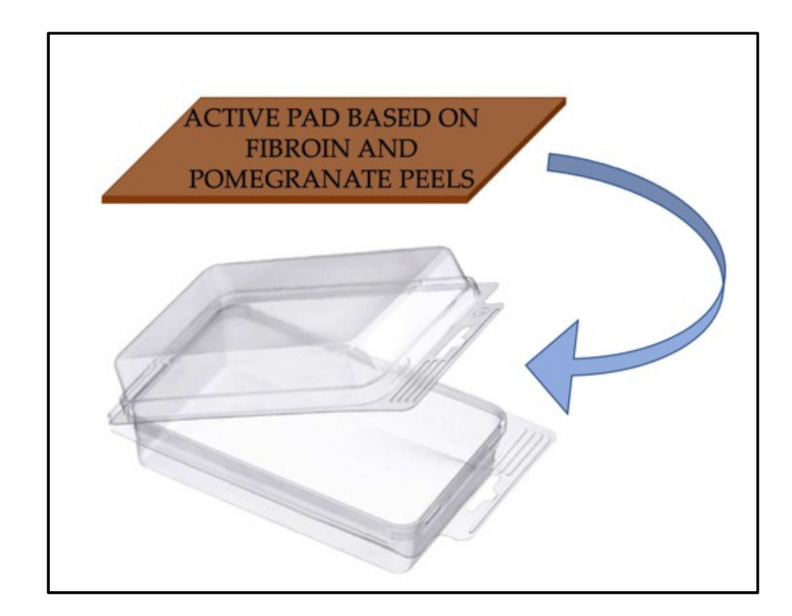
Representation of potential future applications of developed active pad.

On the basis of the above-reported considerations, in the current article, for the first time, an active polymeric system based on fibroin enriched with pomegranate peel powder, was prepared and characterized from structural, morphological, antioxidant and antimicrobial points of view, in comparison to simple pomegranate peel powder. Therefore, the fibroin that represents a safe, natural and edible matrix to incorporate the peel particles, gave rise to an active system. It can be intended as a pad that could find potential future applications as antibacterial/antioxidant system, inserted in other biodegradable food packaging (Figure 1). This active pad must be implemented with the double purpose of creating a system that recovers as much food by-products as possible and that, at the same time, can be effective for preventing the main detrimental phenomena of food decay.

## 2. Materials and Methods

### 2.1. Fibroin Extraction

Regenerated SF aqueous solutions were prepared according to the procedure previously described in the literature [37]. To this aim, *Bombyx mori* silkworm white cocoons (supplied by CREA, Padua, Italy) were used. Briefly, cocoons were degummed in boiling Na_2_CO_3_ solution (0.02 M) (Sigma-Aldrich, St. Louis, MO, USA) for 45 min. SF fibers were then washed three times in Milli-Q water and solubilized in LiBr solution (9.3 M) at 60 °C for 5–6 h. Finally, the SF aqueous solutions were dialyzed (dialysis membranes, MWCO12-14000) against distilled water, centrifuged and stored at 4 °C (regenerated SF solutions ~6 wt/vol %). SF solution has been characterized by SDS-PAGE (see Supplementary Material, Figure S1).

### 2.2. Pomegranate Peel Powder Preparation

Pomegranate fruits (Wonderful variety) were kindly provided by A.P.O. Association (Foggia, Italy). The peel powder was prepared according to the same procedure reported in the study by Panza et al. [19]. Briefly, pomegranates were carefully washed, rinsed and cut to separate the peel from the arils. The by-products were dried in a food dehydrator up to final humidity of 8.77%. The obtained powder was then stored in plastic bags at 4 °C. The peel powder particle size measured by a calibrated sieve was 500 μm.

### 2.3. Active System Preparation

The active pad, at a ratio 30:70 by mass (SF:pomegranate powder), was prepared by blending a 6% *w*/*v* of water SF solution containing 20% *w*/*w* of glycerol vs. SF to induce water insolubility [38] with the suitable amount of pomegranate powder. The active pad was obtained by using a support of polydimethylsiloxane (2 × 2 cm) as substrate. One milliliter of the obtained blend was drop-casted, left to dry at room temperature, and peeled off from the substrate.

### 2.4. Structural and Morphological Characterization

The interactions between pomegranate powder and fibroin were investigated by FTIR spectroscopy, using a Bruker Vertex 70 equipped with a diamond crystal single reflection Platinum ATR accessory. The analysis was conducted in the 4000–400 cm^−1^ region with 128 scans and a resolution of 4 cm^−1^. Optical images were also obtained by an ECLIPSE 80i microscope (Nikon, Tokyo, Japan).

### 2.5. Stability of the Polymeric System

The water stability of the SF-pomegranate active pad was evaluated considering the mass loss, by applying the following equation:

Mass loss (%) = [(m_i_ − m_e_)/m_i_] × 100

where m_i_ is the initial mass and m_e_ is the mass of the pad after being in water at room temperature for 6, 12, 24, 48 and 72 h and then dried at 60 °C in an oven for 2 h. The experiments were performed in triplicate. Specifically, about 0.2 g of active pad were put in water at different times. The collected supernatants were lyophilized and used for ATR measurements.

### 2.6. Antioxidant Activity

The free radical scavenging activity of pomegranate and active fibroin-based pad was evaluated by the 2,2-diphenyl-1-picrylhydrazyl (DPPH) assay [39]. Briefly, 180 µL of pomegranate powder dispersed in water were mixed at different concentrations with 4 mL of DPPH solution (0.1 mM) in 95% ethanol. The same procedure was used for the active pad. The amount of the different samples was varied from 10 to 180 µg in each solution. The absorbance decrease at 516 nm was monitored for evaluating the scavenging activity after storing the sample for 20 min in the dark. The DPPH radical scavenging activity was calculated using the following equation:

I (%) = (1 − (A_sample_ − A_blank_)/A_control_) × 100

where I was the antioxidant activity (%), A_control_ is the absorbance of the DPPH solution, A_sample_ is the absorbance of the sample mixed with the DPPH solution and A_blank_ is the absorbance of each sample without the DPPH solution.

### 2.7. Antimicrobial Properties

To test the antimicrobial activity of both the pomegranate peel powder and the relative active pad, in vitro tests, based on microbial cell growth experiments, were carried out. To this aim, *Pseudomonas fluorescens* and *Pseudomonas putida*, isolated from spoiled fiordilatte cheese, were used as test microorganisms. A cocktail of the two strains was prepared with 1% of each culture, according to that reported in the study of Sportelli et al. [40]. The inoculum was approximately 10^4^ CFU/mL, obtained by diluting the exponentially growing cultures with sterile saline solution (9 g/L NaCl). Direct plate count technique was used to ensure a good degree of reproducibility in the inoculum preparation. For the in vitro tests, PC broth (10 mL) inoculated with the microbial cocktail was spread in different tubes containing the pomegranate peel powder (5% *w*/*w*) or the active pad (0.8 g). To make a comparable antimicrobial test between the peel and the pad loaded with the peel powder, a precise amount of active polymer was set, in order to assure the same content of pomegranate powder in the inoculated broth (peel powder = 5% *w*/*w*). Inoculated tubes with only fibroin pad and tubes without any pad or powder were also prepared as controls. All tubes were incubated at 25 °C for 72 h. After 24, 48 and 72 h, aliquots of 1 mL were taken from each tube for microbiological analyses [40]. All analyses were performed twice on two different samples.

In each broth the pH was also measured using a pH meter (Crison, Barcelona, Spain). Two different samples were used for each measurement.

In order to compare the microbial efficacy of the peel powder and the active pad, the following modified version of the Gompertz equation, previously used in the literature [40], was fitted to the experimental data:

$$\mathrm{Log}\left(N\left(t\right)\right)=\mathrm{Log}\left(N_{\max}\right)-A\cdot \exp\left[-\exp\;\left\{\left[\mu_{max}\;\cdot \;2.71\frac{\lambda -t*\;}{A}\right]+1\right\}\right]+A\cdot \exp\left[-\exp\;\left\{\left[\mu_{max}\;\cdot \;2.71\frac{\lambda -t\;}{A}\right]+1\right\}\right]$$

where N(*t*) is the viable cell concentration (CFU/g) at storage time *t*, *A* is related to the difference between the decimal logarithm of maximum bacterial growth attained at the stationary phase and the decimal logarithm of the initial cell load concentration (CFU/g), μ*_max_* is the maximal specific growth rate, *λ* is the lag time (day), N_max_ is the cell load concentration threshold set to 10^6^ CFU/mL, intended as the microbial count when spoilage defects start to appear on food, t* is the time at which the N(*t*) equals N_max_ (h) and *t* is the time (h).

### 2.8. Statistical Analysis

The significant differences (*p* < 0.05) among the mean values of the fitting parameters were calculated according to a previous statistical approach also reported in the literature (ANOVA and Duncan’s multiple range test with the option of homogeneous groups by STATISTICA 7.1 for Windows, StatSoft, Inc., Tulsa, OK, USA) [40].

## 3. Results and Discussion

### 3.1. Morphological Composition

**Figure 2:**
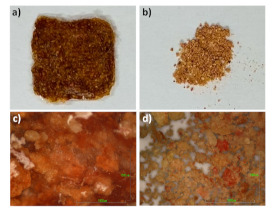
Pictures of active pad (**a**) and pomegranate powder (**b**) and the respective optical images (**c**,**d**).

The active polymeric system (Figure 2A) with a dimension of about 2 × 2 cm, was processed from eco-friendly aqueous solutions. First, we prepared the pad by mixing the SF solution and pomegranate powder (Figure 2B) at 30:70 wt/wt percentage (see experimental part). Glycerol vs. SF (20% *w*/*w*) was also added in order to induce water insolubility of the protein, thus leading to increasing the stability of the resulting SF-pomegranate pad. Next, the blend was drop-casted and left to dry by slow drying procedure. Macroscopically, the pad appears grainy and very dense. Indeed, as indicated by optical images, the powder sample (Figure 2b) shows empty areas (white zones) that are absent in the pad, indicating a more compact structure (Figure 2a) where the fibroin holds the pomegranate powder together acting like a glue.

### 3.2. Structural Characterization

**Figure 3:**
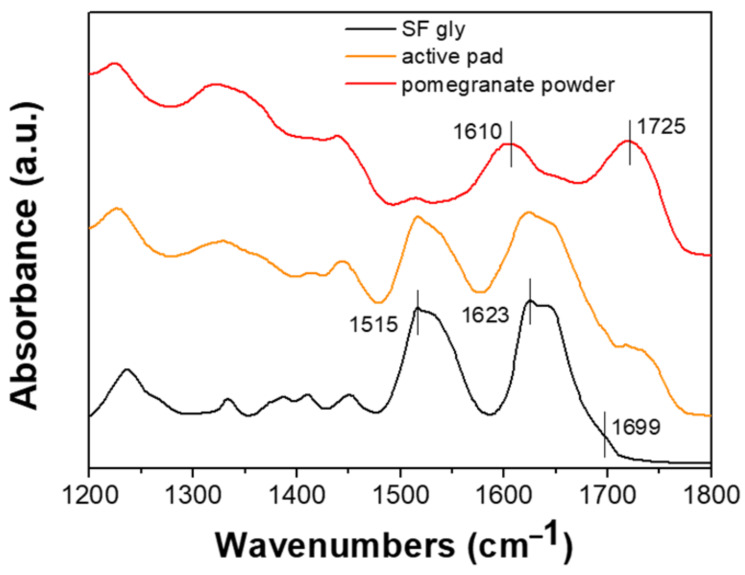
FTIR spectra collected in amide regions (1200–1800 cm^−1^) of SF gly (black line), active pad (orange line) and pomegranate powder (red line).

FTIR experiments were performed in order to investigate the structural properties of SF after blending with pomegranate powder. The spectral region from 1700 to 1500 cm^−1^ is attributed to the absorption of the peptide backbones of amide I (1700–1600 cm^−1^) and amide II (1600–1500 cm^−1^) and thus gives information on the different protein secondary structures [41]. As shown in Figure 3, the FTIR spectra of SF gly (black line) and SF-pomegranate pad (orange line) are mainly characterized by the 1515 cm^−1^ (attributed to the C–C stretching of the aromatic ring and C–H bending of tyrosine in the side chains), 1623 cm^−1^ (intramolecular β-sheets) and 1699 cm^−1^ (intermolecular β-sheets) bands due to the water insoluble silk II conformation, thus suggesting the stability of SF-pomegranate pad in aqueous solution [42]. In addition, the active pad spectrum seems to be the sum of those of pristine SF gly and powder (red line). In comparison with only SF, it shows a much wider amide I band and a new band at around 1725 cm^−1^ due to the pomegranate powder, in particular to the pomegranate peel pectin. The widening of the amide I is probably due to the sum of the pectin band at around 1610 cm^−1^, due to the stretching of COO– carboxylate ion, while the new band at 1725 cm^−1^ is due to the stretching of C=O ester carbonyl groups (red line) [43].

### 3.3. Water Stability

**Figure 4:**
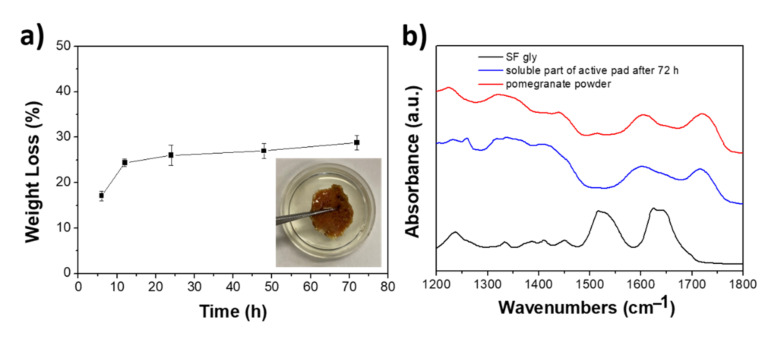
Weight loss in water of SF-pomegranate pad at different incubation times. Inset: picture of the SF-pomegranate pad after 72 h of incubation in water (**a**). Representative FTIR spectrum collected in amide regions (1200–1800 cm^−1^) of the soluble part of active pad (blue line) compared with SF gly (black line) and pomegranate powder (red line) spectra (**b**).

The water stability of SF-pomegranate pad system was evaluated by the weight loss of the samples in water at different times (Figure 4A). As shown in Figure 4A, the weight loss is about 18% after 6 h of incubation, reaching the plateau (weight loss ~28%) after 24 h. FTIR measurements performed on the solubilized part of the film (collected and lyophilized supernatant, Figure 4B, blue line) after 72 h of incubation, indicate that only the active part of the SF-pomegranate pad was released in water. Indeed, the spectrum of the solubilized part is superimposable to that of the powder (red line) and there are no bands (amide I and II) related to fibroin (black line). These results agree with the ATR data listed in the previous paragraph (Figure 3), thus confirming the water stability of SF pad. Notably, the film remained intact even after 72 h of incubation (inset of Figure 4A).

### 3.4. Antioxidant Properties

**Figure 5:**
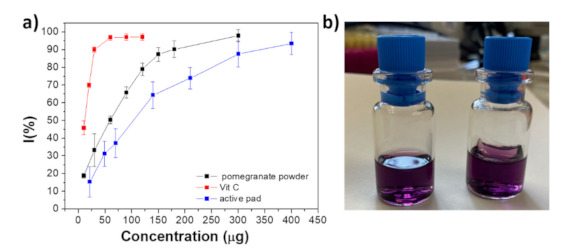
(**a**) DPPH radical scavenging activity of pomegranate powder (black line), SF-pomegranate powder pad (blue line) and vitamin C (red line) measured in the same experimental conditions. (**b**) Pictures of DPPH solution (left) and DPPH solution containing the active pad (right).

In the last few years, several natural antioxidants, such as quercetin, grape seed extract, vitamin C, tannins, oleuropein and rutin, have been incorporated or absorbed into silk fibroin in order to obtain innovative bio-based materials for biomedical applications [44,45,46]. However, only few reports concern the potential application of these SF-antioxidant-based materials as food packaging [47]. Pomegranate peel is characterized by hydrolysable tannins (punicalagin, punicalin, ellagic acid and gallic acid) that are known antioxidants effective in removing radicals [48]. In this respect, the radical scavenging ability of the pomegranate powder used in this study was evaluated in vitro, based on the DPPH assay. Figure 5A shows the scavenging activity of pomegranate powder (black line) compared to that of a well-known antioxidant agent, vitamin C (red line), and SF-pomegranate pad (blue line). Interestingly, the antioxidant activity of pomegranate powder is of the same order of magnitude of that of vitamin C, despite an approximately 6 times lower EC50 value (EC50 pomegranate ~ 60 µg and EC50 Vitamin C ~ 13 µg). As regards SF-pomegranate pad, the EC50 is slightly higher than that of powder alone (~100 µg). This difference cannot be attributed to the presence of fibroin; indeed, pad of SF alone (~31 mg) did not show any antioxidant activity (Figure 5B). After 20 min of incubation, the absorbance of DPPH in presence of SF pad (A = 1.3718) was similar to that of only DPPH (A = 1.3867). Very likely, the slightly higher EC50 of SF-pomegranate pad could be due to the difficulty of homogeneously shattering the pad and, consequently, accurately collecting the antioxidant dispersion. In general, the collected data indicate that pomegranate powder is characterized by a high antioxidant power, comparable to that of vitamin C, therefore, this active system could be considered an alternative antioxidant material to improve the stability of oxidation-sensitive food products.

### 3.5. Antimicrobial Properties

**Figure 6:**
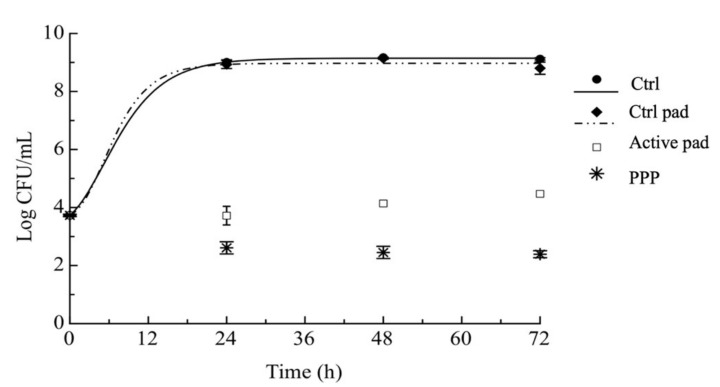
Evolution of *P. fluorescens* and *P. putida* during time. Ctrl: inoculated sample without any pad; Ctrl pad: inoculated sample with fibroin-based pad; Active pad: inoculated sample with fibroin-based pad also containing pomegranate peel powder; PPP: inoculated sample with pomegranate peel powder.

Preliminary tests of antimicrobial efficacy allowed us to find that 5% (*w*/*v*) concentration of peel powder inhibits the growth of *Pseudomonas* spp. (*P. fluorescens* and *P. putida*) (data not shown). Based on these preliminary tests, active films based on sole fibroin and fibroin enriched with fruit by-products were tested for antimicrobial properties. In order to compare the efficacy of the powder with that of the film, 4 pieces of active pads (2 × 2 cm) were used in each tube. The in vitro antimicrobial activity of both powder and active pads was tested in PC broth, inoculated with *Pseudomonas* mix. From results reported in Figure 6, it is possible to infer that during 72 h of monitoring, the two control samples achieved high cell loads, accounting for more than 8 Log CFU/mL just after 24 h. On the other hand, the two active systems, characterized by the sole pomegranate powder and the active pad, maintain very low microbial counts. In particular, these samples highlight a high efficacy against *Pseudomonas* spp., because the powder significantly decreased microbial counts during the observation period. Both active inoculated samples placed in contact with powder or with active pads maintain a microbial concentration around 4 Log CFU/mL during time, that corresponds to the initial inoculation level, thus highlighting the efficacy of peel against spoilage bacteria. It is known that the antimicrobial properties of peel are linked to the high content of polyphenolic compounds, specifically tannic acid equivalents [49,50]. In particular, Grosset-Erard et al. [51] show that the punicalagin contained in pomegranate peels is the main compound responsible for the pronounced antimicrobial activity. Its efficacy has been shown against various Gram-positive and Gram-negative bacteria, also including *Pseudomonas* spp. [51].

The ability of phenolic compounds to inhibit the microbial growth was also recently reported by Kharchoufi et al. [52]. Their results are in agreement with our study, in fact they showed that hydrolysable tannins, present in pomegranate peel extract, reduce *Pseudomonas putida* populations by up to 3.15 Log CFU/mL. In line with our study, Hanani et al. [21] reported that pomegranate peel into fish gelatin film is very effective, but the highest antimicrobial effect was observed on *Staphilococcus aureus*. Furthermore, the study carried out by Ali et al. [53] demonstrated that pomegranate peel powder into starch-based films inhibited the growth of both *S. aureus* and *Salmonella*.

The fitting of the experimental data by the Gompertz equation reported above, also highlighted an important difference between the active systems and the control samples. It is striking to observe that for samples with powder alone and with active pads, the time to reach the microbial threshold was higher than the entire observation period (>72 h) because *P. fluorescens* and *P. putida* never grew in these samples. As a consequence, the trend of data obtained for these two active systems did not allow applying the fitting procedure that was used to only fit experimental data of both control systems. Results from fitting are reported in Table 1 for the two control samples. As can be seen in the table, the two controls are very similar, they show a quite inexistent lag phase (*λ*) and a rapid and high growth rate (μ*_max_*). In addition, t* values (time to achieve 6 Log CFU/mL) of the two control systems are reached after 6.4 h (Ctrl) and 5.9 h (Fibroin pad) of monitoring. Giving this experimental evidence, the active pad demonstrated strong antimicrobial activity against selected target microorganisms.

## 4. Conclusions

Experimental data highlight that for the first time, fibroin was used to realize an edible pad containing pomegranate peel powder rich in bio-active agents. The system is a material with a compact structure and with desired properties, to be intended as an antimicrobial/antioxidant pad to be glued to the bottom of other packaging such as a bottle or a tray. Pomegranate peels represent an abundant by-product among fruit waste and therefore recycling to improve the performance of this pad system could represent a great advantage, as well as from the environmental point of view. In fact, pads with pomegranate peel presented both antioxidant and antimicrobial properties, comparable to that of fruit powder as it is. Even though further study is still necessary to deepen the research topic, current results support the idea that silk fibroin with by-products can be an effective, water-based material that could find further applications. The water-based processing, the comestible nature of silk fibroin and the natural origin of pomegranate peel as active ingredient, makes the approach a promising alternative, more sustainable, for developing active pad systems.

## Figures and Tables

**Table 1:** Values of parameters calculated by fitting the experimental data.

Samples	*A* (Log CFU/mL)	μ*_max_* (ΔLog CFU/mL/h)	*λ* (h)	t* (h)
Ctrl	5.8 ± 0.15 ^a^	0.42 ± 0.03 ^b^	0.2 ± 0.56 ^b^	6.4 ± 0.18 ^a^
Fibroin pad	5.4 ± 1.0 ^a^	0.51 ± 0.7 ^a^	1.3 ± 0.52 ^a^	5.9 ± 2.6 ^a^

^a,b^ Values (means ± SD) marked with different superscript letters in the same column are significantly different (*p* < 0.05). Ctrl: inoculated sample without any pad; Fibroin pad: inoculated sample with fibroin-based pad. *A* is related to the difference between the decimal logarithm of maximum bacterial growth attained at the stationary phase and the decimal logarithm of the initial cell load concentration (CFU/g), μ*_max_* is the maximal specific growth rate, *λ* is the lag time (day), t* is the time at which the N(*t*) equals N_max_ (h).

## Data Availability

The raw data will be made available upon request.

## Supplementary Materials

The following are available online at https://www.mdpi.com/article/10.3390/foods10122921/s1, Figure S1: SDS-PAGE of silk fibroin solution.
