# Supplementary material for: Silk Fibroin and Pomegranate By-Products to Develop Sustainable Active Pad for Food Packaging Applications
Source: Foods. 2021 Nov 25;10(12):2921. doi: 10.3390/foods10122921 (PMC8700627; doi:10.3390/foods10122921)
Supplement: Supplementary file 1 [file foods-10-02921-s001.zip › foods-1437004-supplementary.pdf]

Supplementary Info

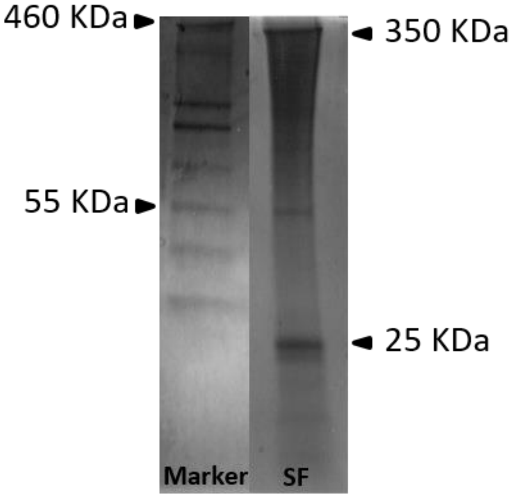

Figure S1: SDS-PAGE of silk fibroin solution.

SDS-PAGE analysis of the SF used in this study recalls that reported in literature for similar SF sample [Biomacromolecules, 2008, 9, 1299; Soft Matter, 2013, 9, 138]. Specifically, regenerated SF aqueous solution (Figure S1) shows a broad band close to 350 kDa and a very weak band at approximately 25 kDa. The former band corresponds to the heavy chains (350 kDa) of silk protein and the latter band at 25 kDa corresponds to the light chain.
